# First Report of Clivus Osteomyelitis Caused by Nocardia veterana in a Lung Transplant Recipient

**DOI:** 10.7759/cureus.36487

**Published:** 2023-03-21

**Authors:** Daphne-Dominique H Villanueva, Guy El Helou

**Affiliations:** 1 Infectious Diseases, West Virginia University, Morgantown, USA; 2 Infectious Diseases, University of Florida, Gainesville, USA

**Keywords:** transplant, sinusitis, skull base, clivus, osteomyelitis, nocardia veterana

## Abstract

*Nocardia* species have been implicated as a cause of pulmonary, cutaneous, ocular, and disseminated central nervous system disease. Dissemination to the bones, commonly the spine, has also been described in the literature. However, isolated osteomyelitis of the skull base is rare. Additionally, advances in the use of molecular techniques have identified many new *Nocardia *species, including *Nocardia veterana* that was thought to be clinically insignificant when it was first identified. Here, we report the clinical features and treatment approach for a lung transplant patient who developed *N. veterana *clivus osteomyelitis secondary to sphenoid sinusitis. It is the first case of skull base osteomyelitis caused by this rare species of *Nocardia*.

## Introduction

*Nocardia *species are filamentous, gram-positive, partially acid-fast, and aerobic actinomycetes that are ubiquitous in the environment [1]. While *Nocardia *species cause disease in immunocompetent hosts, they are often considered opportunistic pathogens especially in patients with cell-mediated immunity defects, such as organ transplant recipients [2,3]. *Nocardia* causes disease either through inhalation causing pneumonia or via the skin causing skin and soft tissue infections. In immunocompromised patients, *Nocardia* can enter the bloodstream seeding the brain and bone. Osteomyelitis is an unusual manifestation. As a result of small patient numbers, only case reports have been published. To the best of our knowledge, only one case of *Nocardia* clivus osteomyelitis has been reported, and this concerned an *N. abscessus *species in a 74-year-old woman who presented with a sinus headache and diplopia [4]. Here, we present the first case of clivus osteomyelitis, a rare type of skull base osteomyelitis, caused by *Nocardia veterana*, a rare species that was first identified in 2001 at a veteran's hospital in Australia and whose pathogenicity is still poorly appreciated.

## Case presentation

**Figure 1:**
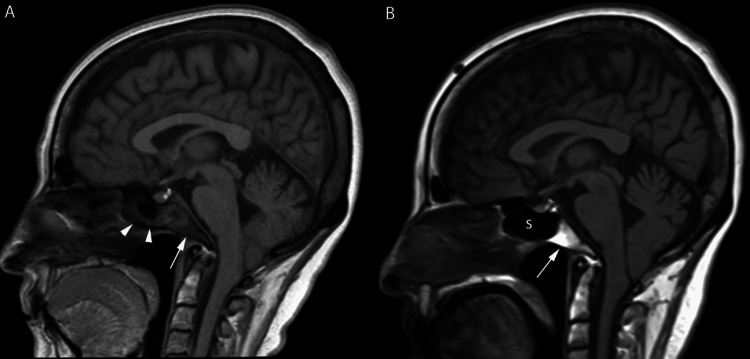
Magnetic resonance imaging of the head Sagittal T1 weighted images (A) demonstrate mild mucoperiosteal thickening in the sphenoid sinus (arrowheads in A) with abnormal signal intensity within the adjacent clivus (arrow in A). The abnormal signal intensity in the clivus becomes more apparent (arrow in B) with no significant sinus disease in the adjacent sphenoid sinus (s in B).

A 61-year-old woman with a history of Sjogren’s disease and bilateral lung transplant for interstitial lung disease was hospitalized one year post-transplant for intractable headaches. The headaches were associated with right-sided facial tenderness that was refractory to analgesics and antibiotics for presumed sinusitis. She was on tacrolimus, azathioprine, and prednisone for immunosuppression; and valganciclovir and dapsone for cytomegalovirus and *Pneumocystis jirovecii *prophylaxis, respectively. Magnetic resonance imaging (MRI) of the brain (Figure 1) demonstrated opacification of the sphenoid sinus with findings concerning clivus osteomyelitis and dural enhancement.

The patient underwent bilateral nasal endoscopy and right endoscopic sphenoidotomy with tissue biopsy. Histopathologic examination demonstrated acute inflammation of the sinus and bone. Five days later, the fungal culture grew partially acid-fast, branching gram-positive rods. This was ultimately identified as *N. veterana* by *hsp65* gene sequencing by the state laboratory. A computed tomography (CT) scan of the chest did not show any pulmonary findings consistent with infection. Empiric therapy was initiated with oral trimethoprim-sulfamethoxazole (TMP-SMX) two double-strength tablets twice daily (the equivalent of 15 mg/kg/day), oral linezolid 600 mg twice daily, and intravenous cefepime two grams every eight hours. Routine monitoring of labs revealed hyperkalemia requiring a lower dose of TMP-SMX adjusted to one double-strength tablet three times daily. She was discharged home on outpatient parenteral antimicrobial therapy (OPAT). On the third week of therapy, routine lab monitoring revealed pancytopenia. This prompted the discontinuation of linezolid, packed red blood cell (PRBC) transfusions, and subcutaneous filgrastim with good response. Based on susceptibility results (Table 1), her antibiotic regimen was tailored to dual therapy with oral TMP-SMX and intravenous cefepime.

**Table 1:** Susceptibility testing results for Nocardia veterana MIC, minimum inhibitory concentration; S, sensitive; TR, tentatively resistant; TS, tentatively sensitive; R, resistant

Antibiotics	MIC mcg/mL	Interpretation
Amikacin	≤8	S
Amoxicillin-clavulanate	>32/16	TR
Azithromycin	≤16	TS
Cefepime	≤4	TS
Cefotaxime	≤8	TS
Ceftriaxone	≤8	TS
Ciprofloxacin	8	R
Clarithromycin	≤0.25	S
Clofazimine	≤0.5	TS
Gentamicin	4	TS
Imipenem	≤2	S
Kanamycin	≤8	TS
Linezolid	≤1	S
Minocycline	≤1	TS
Tobramycin	16	R
Trimethoprim/sulfamethoxazole	≤0.5/9.5	S

The patient reported rapid improvement in headaches as early as four weeks after the initiation of antibiotics. Repeat MRI at that point was already showing improved edema of the clivus, though inflammatory changes consistent with active infection persisted. Five months into her therapy, TMP-SMX was changed to oral minocycline 100 mg twice daily due to hyperkalemia. At six months, MRI showed marked improvement of marrow signal in the clivus consistent with improving osteomyelitis. The same stable finding was noted at nine months and 12 months. Intravenous cefepime and oral minocycline were completed at 12 months. Her symptoms have completely resolved by then.

## Discussion

The hallmark of *Nocardia *infection is the diversity of its clinical presentations in human hosts. It commonly infects the lungs, skin, and brain. One retrospective study of 112 adults with *Nocardia* infection stratified the patients based on host factors: 67 (60%) were immunocompromised, and 45 (40%) were immunocompetent [2]. Sites of infection between groups were compared. The study found that osteoarticular structures were rarely involved and identified only two patients in the immunocompromised group and one patient in the immunocompetent group [2]. In another retrospective review study of 54 solid organ transplant (SOT) recipients with *Nocardia* infection, one patient had bone and lung involvement; and no patient had isolated bone involvement [5]. There are scattered case reports of *Nocardia* osteomyelitis involving the spine secondary to skin lesions or trauma. In one report, a 69-year-old man who was treated for an *N. brasiliensis *skin abscess and underwent multiple incisions and drainage over the course of seven years developed progressive leg weakness. He was later found to have vertebral osteomyelitis and an epidural abscess with *N. brasiliensis *[6]. In another case report, a healthy 21-year-old woman with trauma to the knee complained of gradually worsening pain and swelling of the leg and was found to have femorotibial osteomyelitis due to *N. brasiliensis * [7].

Osteomyelitis involving the clivus, a bone at the base of the skull, is a rare and life-threatening infection. Most cases of skull base osteomyelitis occur secondary to the direct spread from contiguous structures such as untreated malignant otitis externa or less frequently from a paranasal sinus infection. Due to the presence of several important adjacent structures, disease progression can lead to serious complications such as cavernous sinus thrombosis and abducens nerve palsy [8]. Although various pathogens, including *Pseudomonas*, *Staphylococcus*, and mucormycetes, have been implicated as causes of skull base osteomyelitis, to our knowledge, only one case of primary *Nocardia* clivus osteomyelitis has been reported [8,9]. This was a case of an immunocompetent 74-year-old patient who presented with headaches and abducens and hypoglossal nerve palsies [4]. The ability of *Nocardia* to mimic other infections results in delayed diagnosis and can contribute to increased mortality. A high index of suspicion should be maintained with refractory symptoms that do not respond appropriately to antibiotics. Radiological imaging is essential to evaluate the extent of the disease process. Additionally, surgical biopsy with histopathological and microbiological examination should be performed to rule out other diagnoses and to guide appropriate treatment [4,8,9].

While the number of species that make up the genus *Nocardia* is rapidly expanding, the most important causes of infection in transplant recipients are *N. asteroides*, *N. farcinica*, *N. nova*, *N. brasiliensis*, *N. otitidiscaviarum,* and *N. transvalensis *complexes [10]. Molecular methods that rely on 16S ribosomal ribonucleic acid (rRNA) gene sequencing resulted in the identification of previously unknown species, including *N. veterana* named after the veteran’s hospital (Heidelberg, Victoria, Australia) where the organism was first isolated. This species was isolated from a bronchoscopic lavage fluid of a 78-year-old patient with tuberculous pleurisy. Further clinical details were not provided, except that this isolate was thought to be clinically insignificant [11]. Among *Nocardia* species causing infections,* N. veterana* is rarely isolated and is mostly described as a cause of pulmonary infections [12]. Case reports have also described *N. veterana *causing soft-tissue abscess, abdominal infection, mycetoma, brain abscess, and endophthalmitis [13].

Historically, TMP-SMX is selected as the backbone for treatment since it is active in vitro against most *Nocardia *species*. *However, resistance to TMP-SMX is emerging, most likely due to the widespread use of TMP-SMX for *Pneumocystis jirovecii* prophylaxis in immunocompromised patients. In one study that tested the susceptibility of 14 different *Nocardia *species, all isolates of *N. veterana *were susceptible to cefepime, linezolid, imipenem, amikacin, and ampicillin. Only 75% were susceptible to TMP-SMX and ceftriaxone [14]. According to one report, the strain of *N. veterana *showed antimicrobial susceptibility patterns essentially identical to those obtained for *N. nova *and *N. africana* with slight variations in ampicillin minimum inhibitory concentrations [12]. In our case, a multidrug regimen with good bone penetration combining intravenous cefepime and orally administered linezolid and TMP-SMX was selected empirically and then adjusted once sensitivities were available.

The management of *Nocardia* clivus osteomyelitis involves a prolonged antibiotic regimen tailored to the species’ susceptibility profile as well as surgical debridement which seems to improve clinical outcome [7-9]. Despite complete eradication of the infection, cranial neuropathies usually resolve slowly, as was seen in a prior case report [4]. It is worth noting that even after successful medical and surgical intervention, the reported mortality rate of skull base osteomyelitis may be as high as 30% [9]. Another challenge in the field of post-SOT nocardiosis is that combination therapy can be difficult to manage because of toxicity and interactions with immunosuppressive medications. Current recommendations suggest at least six months of antimicrobial therapy for pulmonary or soft tissue infections, six to 12 months for osteomyelitis, and a minimum of nine to 12 months for brain abscess [10,15].

## Conclusions

The present case offers a significant addition to the literature. First, our case constitutes the second report of *Nocardia *species causing sphenoid sinusitis with direct extension into the clivus. This highlights the importance of considering *Nocardia *clivus osteomyelitis in the differential diagnosis of progressive skull base lesions, especially when early intervention is key to the management of this rare and life-threatening disease. Second, our patient developed an infection involving *N. veterana*, a species whose pathogenic potential has not been fully described and which has not been reported before in association with skull base osteomyelitis. This emphasizes the importance of accurate identification of *Nocardia *to the species level because it helps define the spectrum of diseases caused by each species, and it helps guide antibiotic therapy. Third, this case presents the first report of *N. veterana* clivus osteomyelitis in a lung transplant recipient. This population is especially prone to atypical infections, and *Nocardia* infections should always be part of the differential.
